# Supplementary material for: Ion sieving in graphene oxide membrane enables efficient actinides/lanthanides separation
Source: Nat Commun. 2023 Jan 17;14:261. doi: 10.1038/s41467-023-35942-1 (PMC9845371; doi:10.1038/s41467-023-35942-1)
Supplement: Supplementary file 1 — Supplementary Information [file 41467_2023_35942_MOESM1_ESM.pdf]

Supplementary Information

Ion Sieving in Graphene Oxide Membrane Enables Efficient Actinides/Lanthanides Separation

Zhipeng Wang,† Liqin Huang,† Xue Dong, Tong Wu, Qi Qing, Jing Chen, Yuexiang Lu,* Chao Xu*

Institute of Nuclear and New Energy Technology, Tsinghua University, Beijing 100084, China

*Corresponding author. Email: luyuexiang@mail.tsinghua.edu.cn (Y. L.); xuchao@tsinghua.edu.cn (C. X.).

†These authors contributed equally to this work.

1. Supplementary Figures

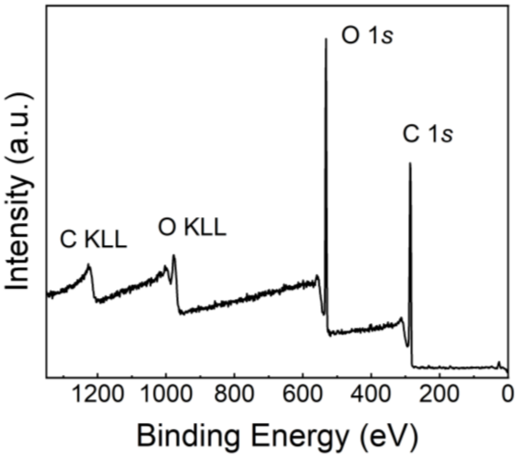

Supplementary Fig. 1 XPS spectra of graphene oxide membrane. The C 1*s* signal of GO consists of three different chemically shifted components which can be deconvoluted into: C=C/C-C in aromatic rings (284.5 eV); C-O (286.5 eV); C=O (288.2 eV).[1] The spectrum of O 1s for GO present two contributions: C=O (530.9 eV); C-O (532.3 eV)[2]. As C and O has relatively close photoelectron kinetic energy, the calculation of C/O ratio is simplified as $n_C/n_O = I_C \cdot \sigma_O / I_O \cdot \sigma_C$, where n_C is the concentration of C atoms, I_C is the photoelectron peak intensity (peak area) of C atoms, σ_C is the photoionization cross section of C atoms. n_O, I_O and σ_O are the corresponding terms for O atoms. The C/O ratio is calculated to be ~2.45 for the GO in our GOM.

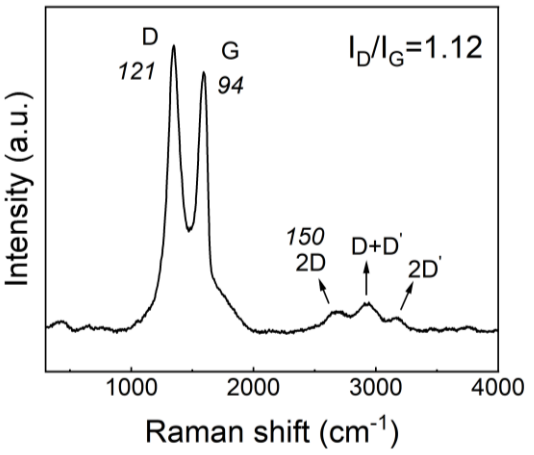

Supplementary Fig. 2 Raman spectra of GOM sample. Full width at half-maximum values are given in italic numbers in the spectrum. The two most intense features are the D peak at 1349 cm^{-1} and the G peak at 1585 cm^{-1}. The G peak is assigned to the doubly degenerate zone center E_{2g} mode.[3] The intensity of the D peak evolves on introducing defects into the lattice.[4] The 2D band at ~2676 cm^{-1} is the second order of zone-boundary phonons.[5] The intensity ratio

of the D peak and the G peak (I_D/I_G) is often used to characterize the degree of defects.[4, 6] By taking the full width at half-maximum (FWHM, Γ) of the D, G and 2D peaks into account, broad peaks of our GO membranes suggest a high degree of defects.

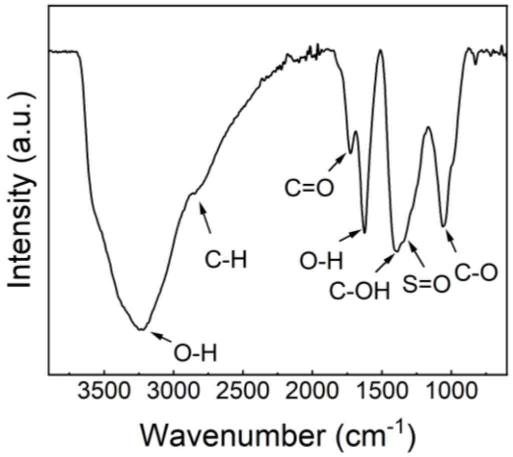

Supplementary Fig. 3 FT-IR spectra of GOM sample. The signals observed between 2200 and 3700 cm^{-1} are due to the O-H stretching vibrations of the water molecules and structural OH groups. Carbonyl-groups can be identified by the absorption at 1725 cm^{-1}. The peak at 1625 cm^{-1} may be assigned to the bending modes of water molecules.[7] The peak at 1400 cm^{-1} refers to the bending of C-OH groups.[8] The peak at 1340 cm^{-1} might be assigned to the stretching modes of covalent sulfate, which is likely brought in during the synthesis process.[9] The peak at 1055 cm^{-1} can be assigned to C-O entities.[7]

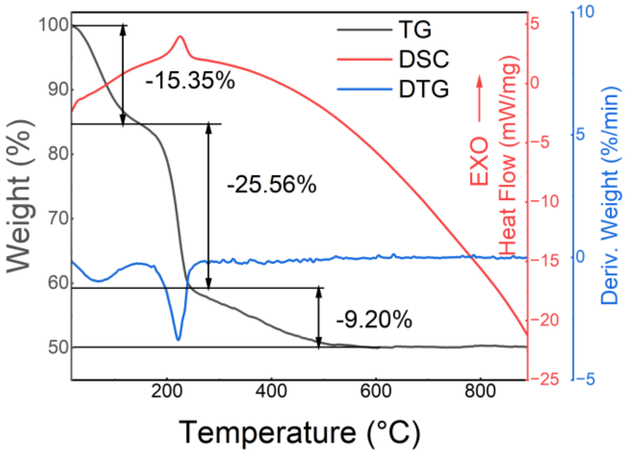

Supplementary Fig. 4 Thermogravimetric analysis (TGA, black curve), differential Scanning Calorimetry (DSC, red curve) and first derivative of the thermogravimetric data (DTG, blue curve) of pristine GO membranes. Heating rate of 5 °C/min, room

temperature to 900 °C in N_2 flow. The thermogravimetric analysis (TGA) curves of GO show three main regions with considerable weight loss. The first weight loss (about 15.35%) from 34 °C (onset temperature) to 115 °C (endset temperature) is mainly caused by dehydration.[10] A rapid weight loss (about 25.56%) occurs in the range of 193 °C (onset temperature) to 237 °C (endset temperature) due to the pyrolysis of the labile oxyen-containing functional groups and the removal of strongly bound water.[10] The DSC signal also shows an apparent exothermic peak at ~225 °C, which may caused by the reduction of graphene oxide.[11] The weight loss lasting up to 486 °C corresponds to the recovery of graphitic order.[12]

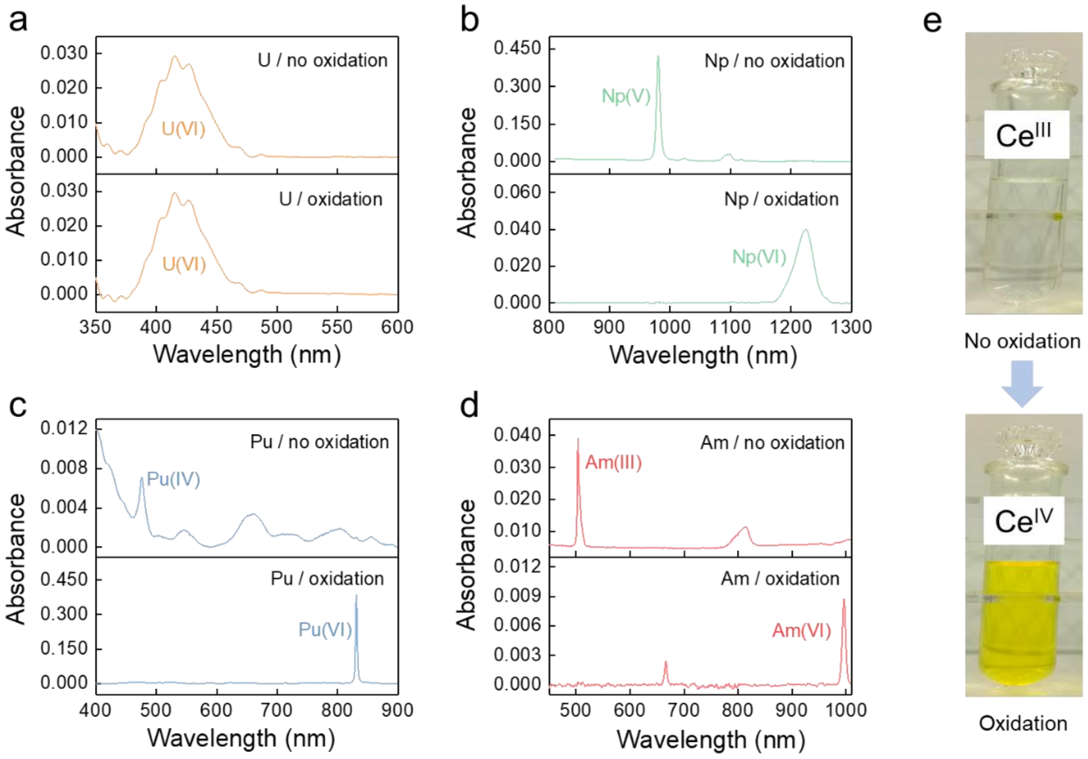

Supplementary Fig. 5 Oxidation of actinides and lanthanides by $NaBiO_3$. UV-Vis-NIR spectra of (a) U, (b) Np, (c) Pu, (d) Am and images of (e) Ce before and after oxidation. For the oxidation, the solution of corresponding metal ions was mixed with sufficient $NaBiO_3$ in 3.0 mol/L HNO_3 for 2 h.

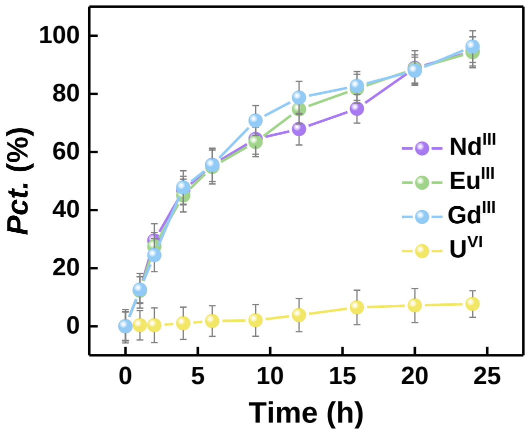

Supplementary Fig. 6 Permeation percentages of lanthanides and uranyl as a function of time. FC: U or Ln solution with $NaBiO_3$ oxidant in 3.0 mol/L HNO_3; RC: 3.0 mol/L HNO_3 and 0.1 mol/L TODGA/*n*-dodecane. All the error bars represent the standard deviation of the experiments.

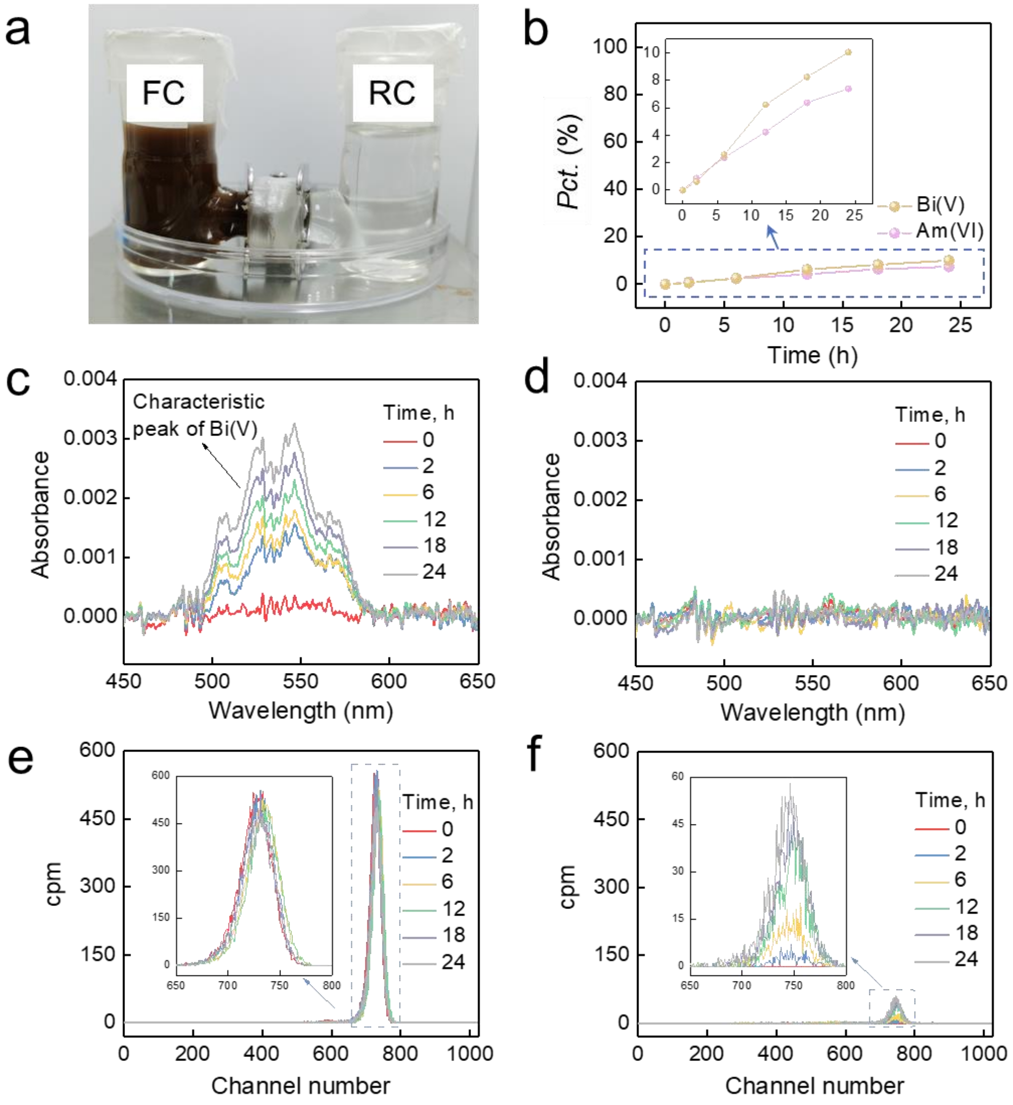

Supplementary Fig. 7 Permeation of Bi(V) and Am(VI) in GOM. (a) Photo of the setup for Am(VI) and $NaBiO_3$ permeation test; (b) Permeation percentages of Bi and Am as a function of time; (c) Absorption spectra of the solution in the FC; (d) Absorption spectra of the solution at the RC; (e) LSC spectra of the solution in the FC; (f) LSC spectra of the solution in the RC. Experimental conditions: (FC) Initial aqueous phase: ~48,000 cpm/mL of Am-241 mixed with 25 g/L $NaBiO_3$ in 3.0 mol/L HNO_3; (RC) Initial aqueous phase: 3.0 mol/L HNO_3.

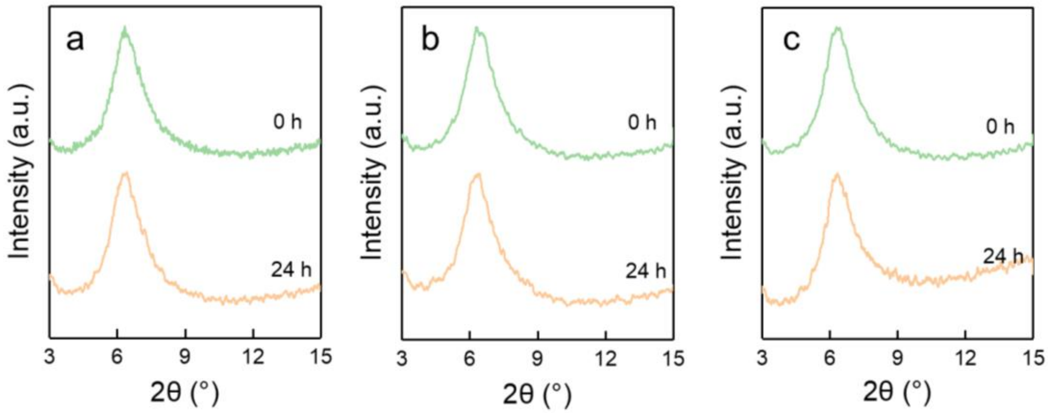

Supplementary Fig. 8 XRD patterns of GOM before and after metal ion permeation for 24 h. The feed solution contains (a) 5 mmol/L U(VI); (b) 5 mmol/L Nd(III); (c) 18 mmol/L Ce(III) + 29 mmol/L Nd(III) + 1 mmol/L Eu(III) + 1 mmol/L Gd(III) (Corresponding to the composition of Ln ions in the simulated feed solution in **Table S2**), $[HNO_3]$ = 3.0 mol/L.

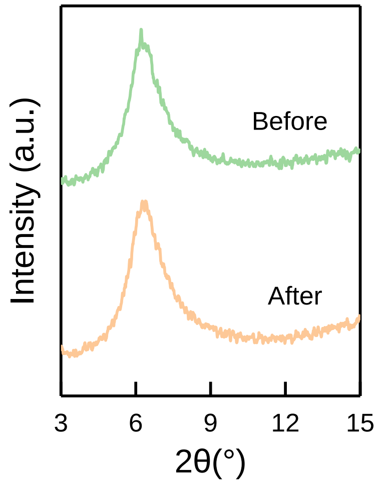

Supplementary Fig. 9 XRD patterns of GOM before and after sieving for 24 h in the presence of $NaBiO_3$. The initial feed solution is 3.0 M HNO_3 solution contains ~15 g/L $NaBiO_3$ solid powder.

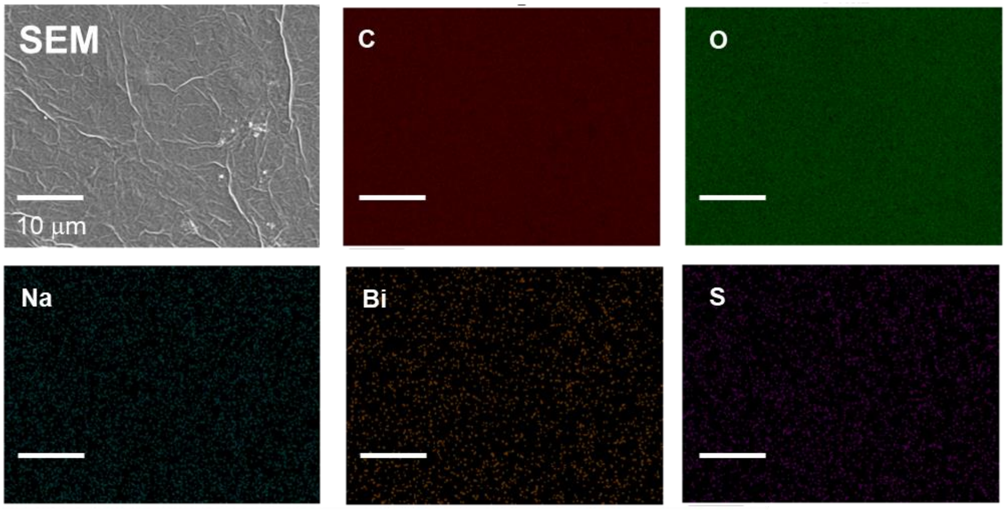

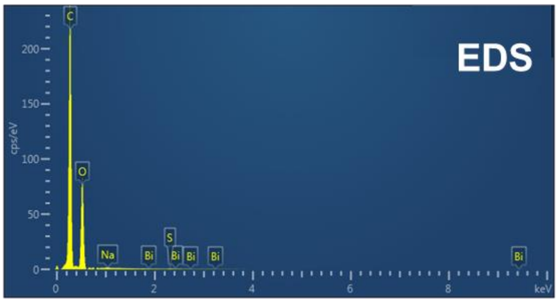

Element	wt%	wt% Sigma
C	70.46	0.21
O	28.92	0.12
Na	0.09	0.02
S	0.42	0.06
Bi	0.11	0.27
Total	100.00	

Supplementary Fig. 10 SEM-EDS analysis results of GOM surface after permeation test in the presence of solid $NaBiO_3$. Obivously, the amount of Bi and Na on the GOM surface is negeligible.

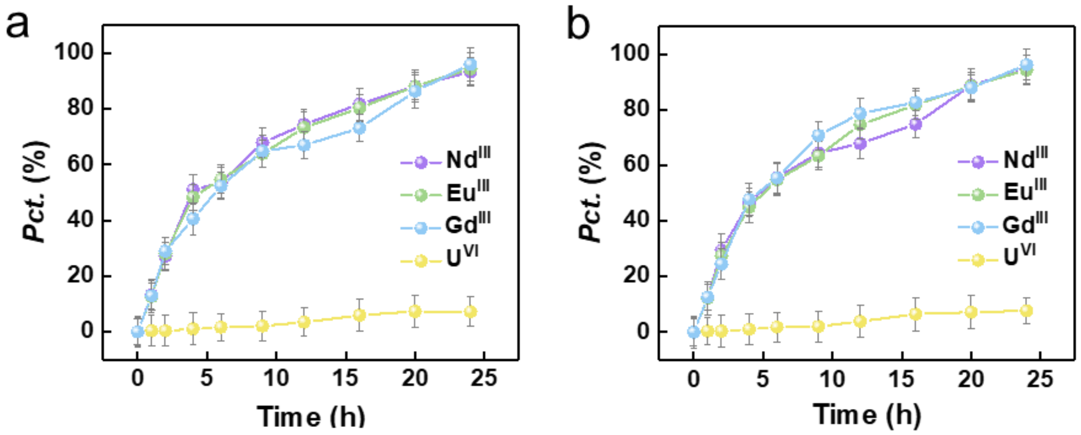

Supplementary Fig. 11 Comparison of ion sieving of Nd(III), Eu(III), Gd(III) and U(VI) through GOM. (a) With and (b) without the presence of $NaBiO_3$ in the feeding solution. (Data are extracted from Figure 3 in the main manuscript). The similar permeation behaviors of these ions indicate that $NaBiO_3$ has no apparent influence on the permeation of Ln/An ions that are inert to oxidation. All the error bars represent the standard deviation of the experiments.

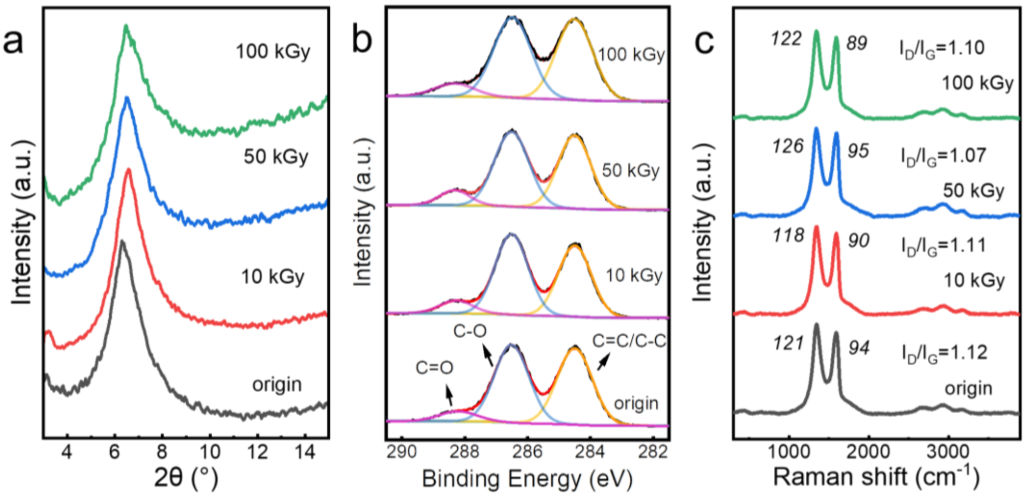

Supplementary Fig. 12 Characterization of GOM before and after gmma-irradiation. (a) XRD patterns, (b) Raman spectra and (c) XPS spectra of gamma-irradiated (10, 50, 100 kGy) and un-irradiated GOM. The dose rate is 120 ± 5 Gy/min. All the GOMs were irradiated in 3.0 M HNO_3. The almost identical features of XRD, Raman, and XPS results prove the stability of GOM against gamma-irradiation under highly acidic conditions.

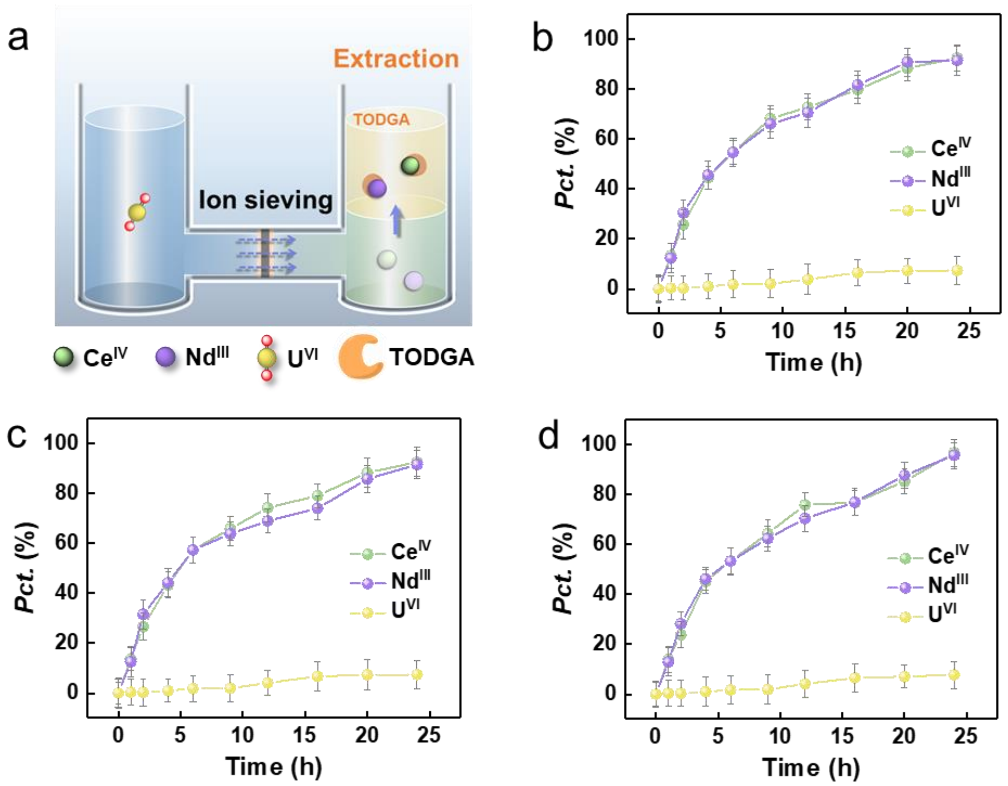

Supplementary Fig. 13 Sieving of Ce(IV), Nd(III) and U(VI) in 3.0 mol/L HNO_3 as a

function of time through gamma-irradiated GOM of different dose. (a) Diagram of sieving setup; (b-d) GOM with irradiation dose of 10 kGy, 50 kGy and 100 kGy, respectively. Initial concentration of Ce, Nd and U is around 7.1 mmol/L, 6.9 mmol/L and 4.2 mmol/L, respectively. All the error bars represent the standard deviation of the experiments.

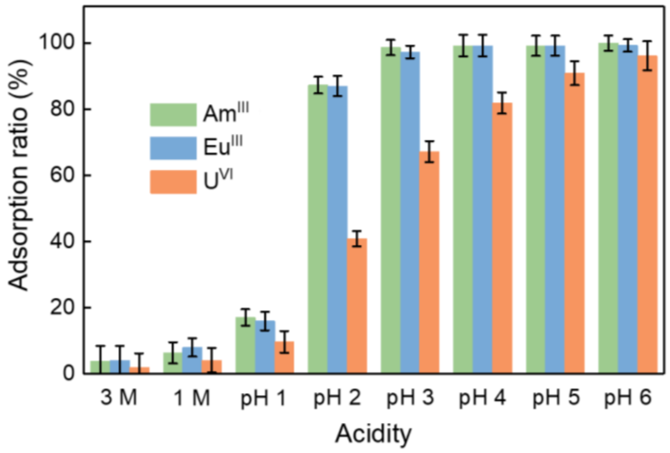

Supplementary Fig. 14 Adsorption of ^{241}Am(III), ^{152,154}Eu(III) and U(VI) by the GO sheets under different aqueous acidities. Initial concentrations of ^{241}Am(III), ^{152,154}Eu(III) and U(VI): ~10000 α cpm/mL, ~10000 β cpm/mL and 4.2 mmol/L, respectively. Solid (GO)/liquid ratio: 1 mg GO/ 10 mL aqueous solution. Adsorption time: 24 h. The adsorption of the three ions by GO is negligible in 3.0 M HNO_3. All the error bars represent the standard deviation of the experiments.

2. Supplementary Tables

Supplementary Table 1. Variation of the total organic carbon (TOC, ppm) in the FC as a function of time. Experimental conditions: (FC) 3.0 mol/L HNO_3; (RC) 0.1 mol/L TODGA/*n*-dodecane and 3.0 mol/L HNO_3. The results suggest no TODGA or *n*-dodecane has permeated through the GOM from the RC to FC, thus avoiding direct contact of high valent actinides with these organic reagents.

Time (h)	TOC (ppm)
0	1.57 ± 0.10
6	1.61 ± 0.10
12	1.63 ± 0.10
18	1.59 ± 0.10
24	1.65 ± 0.10

Supplementary Table 2. Composition of the mixed actinides/lanthanides waste solution.

Element or isotope	^{238}U	^{237}Np	^{239}Pu	^{241}Am
Concentration (g/L)	0.502	~2.34E-4	~4.73E-6	~2.42E-7
Element or isotope	Ce	Nd	Eu	Gd
Concentration (g/L)	2.469	4.190	0.155	0.142
[HNO_3] = 3.0 mol/L				

3. Supplementary Note 1

For the size of hydrated spheric metal ions in aqueous solution, there are not too much disputes and the values are mainly obtained through measuring the conductivity/diffusion rate or electromigration velocities and then being calculated according to the Stokes' law.[13, 14, 15] Generally, the diameter of hydrated trivalent lanthanides is in the range of 9.0 – 9.3 Å, which indicates that the boundary of these hydrated ions stretches apparently beyond the first hydration shell of the lanthanides. Moreover, the diameter of hydrated trivalent lanthanides exhibits a monotonically increasing trend, which is opposite to the trend of lanthanide contraction effect (in similar coordination environment). This is because the higher charge density of heavier lanthanide makes it attract more outer-coordinated water molecules in the second hydration shell.

However, the exact size of the hydrated actinyl ions (with contributions from outer-sphere waters) has been rarely investigated in the same manner as the lanthanides in literatures. In this work, to estimate the hydration size of actinyl ions, we have consulted literatures dealing with the bulk hydration issue of actinyl ions in aqueous solution.[16, 17, 18, 19] Molecular dynamics (MD) results suggest that the overall hydration structure of UO_2^{2+} is strongly anisotropical, coupling of a conventional hydration sphere in the equatorial region with clathrate-like caps around the axial region, with the second hydration shell of uranyl ion obviously stretches further along the axial direction due to the presence of two yl-O atoms. Generally, the distance of shell boundary from U in the axial direction is about 1.2 times longer than that in the equatorial direction. If we assume the ionic diameter along the equatorial direction is similar to that of trivalent lanthanide ions (this is reasonable because the U-O bond distance in the equatorial plane is close to that of Ln-O bond distance in similar coordination environment), then the overall size of the uranyl ion will be about 1.2 times larger than that of the lanthanide ions. Consequently, the diameter of the uranyl ion can be estimated to be >10.8 Å (The ionic size of U(III) and Ce(III) is similar and the hydrated diameter of Ce(III) is 9.02 Å). For other actinyl ions such as Np(VI), Pu(VI), and Am(VI), we hypothesize that the sizes of their hydrated ions are slightly larger than U(VI) if there is a similar trend in hydrated ion size across the actinyl series as that across the lanthanide series. Another way to estimate the size of hydrated actinyl ions is to roughly add the length of O=An=O to the corresponding hydrated spherical ions. This will result in a much larger size of the actinyl ions, i.e., ~ 3.4 Å larger than the

spherical ions. However, this way is not that reasonable because the hydration behavior of the yl-O atoms in the actinyl ion is very different than that of the central metal.

Moreover, since our permeation tests were conducted in 3.0 M HNO_3 solutions, the presence of a large amount of nitrate ions would also affect the chemical status of the lanthanide and actinide ions. For example, the uranyl ion would form 1:1 complex with nitrate ions in nitric acid solution and this issue has been investigated in a number of literatures.[20, 21, 22] Based on a recently reported $\log\beta$ value of -0.62, we could estimate that ~40% of U(VI) ions will be complexed with nitrate ion while most (60%) of U(VI) ions are in the free hydrated form for ~4.2 mM U(VI) in 3.0 M HNO_3 (the starting conditions for permeation test in our work). There are no reliable data for the stability constants for the complexes of Np(VI), Pu(VI) and Am(VI) with nitrate ion in aqueous solution. But considering the chemical similarity of these hexavalent actinyl ions to U(VI), it is reasonable to assume their stability constants are at the same level as that of U(VI) and thus an appreciable portion of Np(VI), Pu(VI) and Am(VI) may also exist as 1:1 nitrate complex in the solution. The stability constants for the complexation of Am(III) and some of the lanthanides (such as Nd(III)) with nitrate ion are also available in literatures.[23, 24] In general, the complexation of trivalent actinide and lanthanide ions with nitrate ion is slightly stronger than that of uranyl ion. A simple calculation suggests that >50% of the trivalent actinide and lanthanide ions will be complexed with nitrate ion in 3.0 M HNO_3. Upon complexation of nitrate ions, the size of metal complexes will change as compared to the hydrated ions. However, the detailed influence imposed by nitrate complexation on the ions' size is not easy to illustrate. The nitrate ion can complex with the metal ions either in monodentate or in bidentate mode, and the number of water molecules replaced in these two situations will be different. Moreover, the complexation with nitrate is expected to cause obvious disturbance to the outer hydration shell of the metal ions and thus affect their effective size in the solution. In general, we believe complexation with nitrate ions may increase the size of the metal ions due to the relatively large size of nitrate ions than water molecules. However, to fully address these issues, great efforts must be required at both the experimental and theoretical levels. Currently, we expect the complexation of nitrate ion may impose similar effect on the size of trivalent lanthanides and actinyls, because these metal ions only form 1:1 complex with the nitrate ion under our experimental conditions. But for tetravalent lanthanides or actinides such as Ce(IV) and Pu(IV), the impact of nitrate complexation will be more significant due to the strong complexation and the

presence of complexes with high complexation stoichiometry (1:2). And this greater impact has indeed been demonstrated by our experimental results, i.e., the permeation rate of both Ce(IV) and Pu(IV) is apparently slower than that of trivalent lanthanides in natural permeation without the presence of TODGA extraction in the receiving solution (Figure 3b in the manuscript).

4. Supplementary references

1. Hontoria-Lucas C., López-Peinado A. J., López-González J. d. D., Rojas-Cervantes M. L. & Martín-Aranda R. M. Study of oxygen-containing groups in a series of graphite oxides: Physical and chemical characterization. *Carbon* **33**, 1585-1592 (1995).
2. Desimoni E., Casella G. I., Morone A. & Salvi A. M. XPS determination of oxygen-containing functional groups on carbon-fibre surfaces and the cleaning of these surfaces. *Surf. Interface Anal.* **15**, 627-634 (1990).
3. Tuinstra F. & Koenig J. L. Raman Spectrum of Graphite. *J. Chem. Phys.* **53**, 1126-1130 (1970).
4. Ferrari A. C. & Robertson J. Interpretation of Raman spectra of disordered and amorphous carbon. *Phys. Rev. B* **61**, 14095-14107 (2000).
5. Ferrari A. C. & Basko D. M. Raman spectroscopy as a versatile tool for studying the properties of graphene. *Nat. Nanotechnol.* **8**, 235-246 (2013).
6. Lucchese M. M., *et al.* Quantifying ion-induced defects and Raman relaxation length in graphene. *Carbon* **48**, 1592-1597 (2010).
7. Mermoux M., Chabre Y. & Rousseau A. FTIR and ^{13}C NMR study of graphite oxide. *Carbon* **29**, 469-474 (1991).
8. Szabó T., Berkesi O. & Dékány I. DRIFT study of deuterium-exchanged graphite oxide. *Carbon* **43**, 3186-3189 (2005).
9. Petit C., Seredych M. & Bandosz T. J. Revisiting the chemistry of graphite oxides and its effect on ammonia adsorption. *J. Mater. Chem.* **19**, (2009).
10. Eigler S., Dotzer C., Hirsch A., Enzelberger M. & Müller P. Formation and Decomposition of CO2 Intercalated Graphene Oxide. *Chem. Mater.* **24**, 1276-1282 (2012).
11. Jankovský O., *et al.* Reducing emission of carcinogenic by-products in the production of thermally reduced graphene oxide. *Green Chem.* **18**, 6618-6629 (2016).
12. Yang D., *et al.* Chemical analysis of graphene oxide films after heat and chemical treatments by X-ray photoelectron and Micro-Raman spectroscopy. *Carbon* **47**, 145-152 (2009).
13. Nightingale E. R., Jr. Phenomenological Theory of Ion Solvation. Effective Radii of Hydrated Ions. *J. Phys. Chem.* **63**, 1381-1387 (1959).
14. Lundqvist R., Hulet E. K. & Baisden P. A. Electromigration method in tracer studies of complex chemistry .2. Hydrated radii and hydration numbers of trivalent actinides. *Acta Chem. Scand. A* **35**, 653-661 (1981).
15. Tansel B. Significance of thermodynamic and physical characteristics on permeation of ions during membrane separation: Hydrated radius, hydration free energy and viscous effects. *Sep. Purif. Technol.* **86**, 119-126 (2012).
16. Hagberg D., Karlstrom G., Roos B. O. & Gagliardi L. The coordination of uranyl in water: A

combined quantum chemical and molecular simulation study. *J. Am. Chem. Soc.* **127**, 14250-14256 (2005).

17. Chopra M. & Choudhury N. Effect of Uranyl Ion Concentration on Structure and Dynamics of Aqueous Uranyl Solution: A Molecular Dynamics Simulation Study. *J. Phys. Chem. B* **118**, 14373-14381 (2014).
18. Kerisit S. & Liu C. Structure, Kinetics, and Thermodynamics of the Aqueous Uranyl(VI) Cation. *J. Phys. Chem. A* **117**, 6421-6432 (2013).
19. Pérez-Conesa S., Torrico F., Martínez J. M., Pappalardo R. R. & Sánchez Marcos E. A hydrated ion model of [UO2](2+) in water: Structure, dynamics, and spectroscopy from classical molecular dynamics. *J. Chem. Phys.* **145**, 224502 (2016).
20. Rao L. & Tian G. Thermodynamic study of the complexation of uranium(VI) with nitrate at variable temperatures. *J. Chem. Thermodyn.* **40**, 1001-1006 (2008).
21. Suleimenov O. M., Seward T. M. & Hovey J. K. A Spectrophotometric Study on Uranyl Nitrate Complexation to 150 °C. *J. Solution Chem.* **36**, 1093-1102 (2007).
22. Guilbaud P. & Wipff G. Hydration of uranyl (UO_2^{2+}) cation and its nitrate ion and 18-crown-6 adducts studied by molecular dynamics simulations. *J. Phys. Chem.* **97**, 5685-5692 (1993).
23. Rao L. & Tian G. Complexation of Lanthanides with Nitrate at Variable Temperatures: Thermodynamics and Coordination Modes. *Inorg. Chem.* **48**, 964-970 (2009).
24. Tian G. & Shuh D. K. A spectrophotometric study of Am(III) complexation with nitrate in aqueous solution at elevated temperatures. *Dalton Trans.* **43**, 14565-14569 (2014).
